# The great Texas COVID tragedy

**DOI:** 10.1371/journal.pgph.0001173

**Published:** 2022-10-20

**Authors:** Peter J. Hotez

**Affiliations:** 1 Departments of Pediatrics and Molecular Virology and Microbiology, Texas Children’s Hospital Center for Vaccine Development, National School of Tropical Medicine, Baylor College of Medicine, Houston, TX, United States of America; 2 James A Baker III Institute of Public Policy, Rice University, Houston, TX, United States of America; 3 Hagler Institute of Advanced Study and Scowcroft Institute of International Affairs, Texas A&M University, College Station, TX, United States of America; 4 Department of Biology, Baylor University, Waco, TX, United States of America; 5 PLOS Neglected Tropical Diseases, San Francisco, CA, United States of America; PLOS Global Public Health and McGill University, CANADA and Catherine Kyobutungi, PLOS Global Public Health and APHRC, KENYA

The United States of America leads all high-income nations in COVID-19 deaths, even though as a nation at had the greatest access to antiviral vaccines and therapeutics. To understand this disconnect we can look to the COVID-19 deaths and disability in the State of Texas. Because of COVID-19, Texas is enduring one of the greatest human tragedies in its 186-year history. It did not have to be this way.

According to Texas Department of State Health Services and the major publicly accessible disease trackers from Johns Hopkins University, the University of Washington-Institute for Health Metrics and Evaluation (UW-IHME), and *The New York Times*, the number of deaths from COVID-19 in my state of Texas has reached 90,000, as of September 1, 2022 [1–4]. These numbers place Texas just behind California (by only 3,000–4,000) as the US state with the most COVID deaths, but with a Texas population estimated at 29 million compared to 40 million people living in California the proportion of Texas deaths is far higher. Other comparisons—the nation of Canada with 36 million people suffered an estimated 43,000 deaths, while Australia with 25 million experienced 12,000 deaths. It is also worth noting that Texas, Canada, and Australia also share economies of roughly the same size, with a gross domestic product between $1–2 trillion.

**Fig 1:**
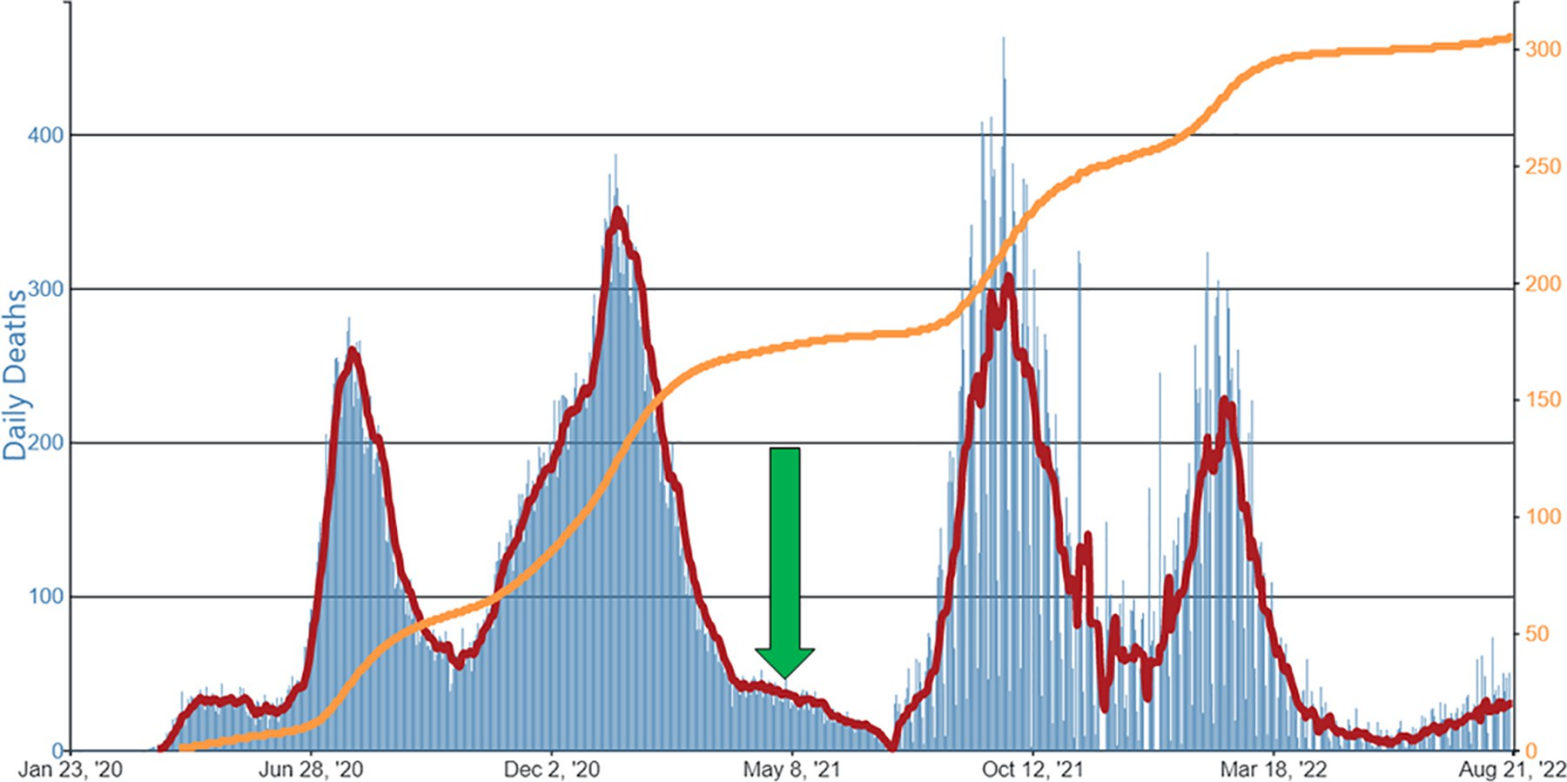
“Daily Trends in Number of COVID-19 Deaths in Texas Reported to the CDC, per 100,000 population”, 2020–2022. Modified from: Centers for Disease Control. https://covid.cdc.gov/covid-data-tracker/#trends_dailydeaths_totaldeathsper100k_48. Accessed August 24, 2022. The arrow marker points to May 1, 2021 when COVID-19 vaccinations became widely available. Orange line is cumulative deaths per 100,000 population.

To understand why Texas suffered 2–3 times more deaths compared to nations of similar populations and economies, we can look to the pattern of Texas deaths during the pandemic. By May 1, 2021, the Biden White House had made it possible for every adult American to receive a COVID-19 vaccination [5]. However, as shown in Fig 1, the COVID-19 deaths in Texas continued even after vaccines were widely available.

In fact, approximately 40,000 of the 90,000 COVID-19 deaths in Texas occurred after May 1, 2021, when any American who wished to take COVID vaccine could do so [3]. Data from the Texas Department of State Health Services (Texas DSHS) reports that 85% of COVID-19 deaths in Texas in 2021 occurred among the unvaccinated [6], while in the first three months of 2022 during the omicron wave the CDC finds rates of death in the US were 20 times higher among unvaccinated people compared to people who were vaccinated and had received a booster [7]. Therefore, the vast majority of the 40,000 deaths occurred among the unvaccinated. To put these numbers in perspective, just over 20,000 Texans lost their lives in World War II, while 6,000 died in the 1900 Galveston storm and flood. Approximately 4,000 Texans die annually from either gun deaths or road traffic annually, and 1,300 Texans died in its worst indigenous war, the Battle of Medina in 1813.

The fact that almost 40,000 Texans might have lost their lives because they refused a COVID-19 vaccination is unique–and no accident. Multiple analyses identify a strong political divide over the acceptance COVID-19 immunizations and death rates, with vaccinations the lowest and death rates the highest in the conservative or “red” states and counties [8]. The term “red COVID” has been invoked to understand this phenomenon [8]; it reflects the strong antivaccine activism promoted by elected officials on the far right and spread on conservative news and social media sites [9]. The rhetoric derives from right wing politics around “health freedom”, both a framework and propaganda tool, which accelerated in Texas in the 2010s for childhood vaccination mandates in schools [10]. According to the Texas DSHS, even as late as September 1, 2022, in many if not most counties in Central Texas and the Panhandle as well as East Texas–all conservative areas of the state—the rates of “fully vaccinated” for adults remain below 50%. These numbers are well below national averages. By encouraging Texans to refuse COVID-19 vaccinations, health freedom propaganda has emerged as a deadly social force. Now, there is evidence that antivaccine activism arising out of Texas could spread internationally to affect both COVID-19 and childhood vaccination rates globally [11].

To be sure, even before COVID-19 vaccines became available many Texans died. In 2020 and early in 2021 some of the highest death rates occurred in working class Hispanic and other minority neighborhoods [12]. This occurred because the essential nature of their work prevented many Hispanic families from isolating at home, in addition to the fact that many lived in multigenerational households. I testified to the US Congressional Hispanic Caucus about “historic decimation” of Hispanic communities in Texas and the US [13]. A subsequent Rice University COVID-19 analysis using data from 2020 found that both Black and Hispanic minority status was “highly correlated with a higher probability of death from the disease” [14].

The disproportionate deaths in Texas among Hispanic and African American groups in 2020 (and possibly thereafter) followed by conservatives who refused a COVID-19 immunization in 2021–22 will leave a haunting legacy for Texas. We will also need to address the specter of long COVID neurologic and cardiovascular declines among the millions of Texans who acquired COVID-19 infection, with the recognition that the risk of long COVID-19 increases among those who experiences severe illness. Once again, the unvaccinated will likely bear the brunt of the long COVID disease burden.

In Texas, many if not most of those who died from COVID-19 in the last half of 2021 and into 2022 could have been saved through immunization. Instead, they became victims of antivaccine activism and aggression that now predominates—for years Texas has been ground zero for the antivaccine health freedom movement in America. Another grim reality: It is doubtful that antivaccine activism will dissipate with the end of the COVID-19 pandemic. Instead, many signs point to its spillover to all childhood vaccines leading to significant protests and future legislative action against Texas school immunization mandates [15].

There is also a paradox. Texas is a state of great universities and the home of the Texas Medical Center—the world’s largest academic medical center where so many biomedical innovations first began. Yet, in this case the dark forces in the state that rail against science and innovation could triumph over biomedicine. We need to understand better the drivers of Texas anti-science aggression and work to diffuse it ahead of the next pandemic that will surely come.
